# A study of the clinical usefulness of the eMEMO®

**DOI:** 10.1002/joa3.70137

**Published:** 2025-07-09

**Authors:** Hinako Nakayama, Nobuhisa Watanabe, Misa Tsukamoto, Miyu Mori, Eri Nakakubo, Shogo Watanabe, Manabu Taniguchi

**Affiliations:** ^1^ Department of Medical Technology Graduate School of Health Sciences, Okayama University Okayama Japan; ^2^ Division of Medical Support Okayama University Hospital Okayama Japan; ^3^ Taniguchi Heart Clinic Fukuyama Japan; ^4^ Faculty of Health Science, Okayama University Okayama Japan

**Keywords:** arrhythmia, atrial fibrillation, ECG, external loop recorder, skin condition

## Abstract

**Background:**

Taking advantage of the characteristics of the novel compact external loop recorder (ELR; eMEMO®), which is waterproof and has an automatic arrhythmia detection and monitoring function for up to 14 days, we investigated the usefulness of ELR measurement, especially during bathing.

**Methods:**

We included 294 patients (102 males and 192 females) who presented with palpitations as their main complaint from April 2021 to September 2022, with a mean age of 57.0 ± 17.4 years. The average wearing period of the ELR was 4.5 ± 0.9 days.

**Results:**

ELR for longer than 24 h led to the first arrhythmia detection in 170 (21.3%) of the 799 cases of all detected arrhythmias, including 11 with premature atrial contraction, 50 with premature ventricular contraction, 8 with atrial fibrillation, 32 with supraventricular tachycardia, and 21 with sick sinus syndrome. They had low noise contamination during measurement. Arrhythmic events during bathing were observed in 52 patients, effectively detecting arrhythmia during bathing, which was difficult with the usual 24‐h Holter electrocardiography (ECG). In addition, the itching and rash of the skin at the electrode attachment over a period of up to 5 days were very mild, indicating the comfort of the waterproof patch‐type electrode.

**Conclusions:**

The patch‐type ELR (eMEMO®) with waterproof design is capable of continuous recording regardless of restrictions such as bathing or exercise and is useful for detecting stable arrhythmias in patients with palpitations.

## Graphical abstract

Taking advantage of the characteristics of the novel compact external loop recorder (ELR; eMEMO®), we investigated the usefulness of ELR measurement. As a result, the patch‐type ELR, which is waterproof and can continuously record regardless of restrictions such as bathing or exercise, is useful for detecting stable arrhythmias in patients.

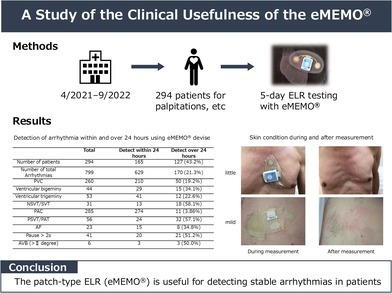

## 1 INTRODUCTION

Arrhythmias are often asymptomatic and may be associated with adverse events such as stroke or peripheral arterial embolism, which can be effectively prevented with anticoagulant treatments if detected early.^1^ However, because some arrhythmias can be difficult to detect because of their short duration, infrequent attacks, and potential lack of any symptoms, proper diagnosis has been difficult.^2^, ^3^ Therefore, it is essential to improve the effectiveness of arrhythmia monitoring. Recently, modalities for ECG monitoring, such as the conventional 24‐h Holter ECG and implantable loop recorder (ILR), have been utilized for arrhythmia detection,^4^ but each has its own advantages and disadvantages; therefore, these modalities must be used accordingly. For example, 24‐h Holter ECG, which is commonly used in routine medical care, has difficulties in capturing palpitations efficiently because of the low frequency of atrial fibrillation (AF) attacks.^5^, ^6^ Additionally, manually triggered extracorporeal event loop recorders may miss asymptomatic or nighttime arrhythmias.^7^ Very long‐term ILR (approximately 2 years) has no restrictions, such as removal during bathing or exercise, and has very high sensitivity for detecting arrhythmias^8^, ^9^; however, this type of ILR needs to be implanted subcutaneously, making it impossible to apply in routine medical care.

Based on this background, we focused on eMEMO®, a new external event loop recorder approved in Japan in 2016, to enable more effective detection of arrhythmia in routine medical care. eMEMO® has some characteristics including: (1) continuous recording for up to 14 days, (2) no need for removal when taking a bath or shower, (3) small size and light weight (25 g), and (4) patch‐type electrode. Although eMEMO® can be an excellent tool for arrhythmia detection suitable for routine medical care, its usefulness in actual clinical practice has not been verified in detail. In particular, fluctuations in the autonomic nervous system during bathing can easily cause Neurally Mediated Syncope (NMS), as well as heart rate and blood pressure changes.^10^, ^11^ In this study, we investigated the usefulness of the device from functional and clinical perspectives in 294 patients with chest symptoms who visited Taniguchi Heart Clinic, focusing on arrhythmia detection during bathing, which is an advantage of the eMEMO®.

## 2 SUBJECTS AND METHODS

### 2.1 Study design and patient selection

This study was conducted at Taniguchi Heart Clinic (Hiroshima, Fukuyama, Japan). This study conformed to the principles outlined in the Declaration of Helsinki regarding research on human subjects and the procedures of the Okayama University Medical Ethics Committee. This was a retrospective study with an opt‐out approach. Between April 2021 and September 2022, 294 patients aged >15 years were examined for chest symptoms. We investigated all patients' medical histories, and routine ECG, long‐term ELR testing with eMEMO®, chest X‐rays, and periodic clinical examinations were conducted. Arrhythmia was diagnosed by a cardiologist based on medical data. In this experiment, the continuous recording period of eMEMO® was set to 5 days in consideration of patient burden. The patient had the right to discontinue wearing the electrode at any time if they experienced discomfort or difficulty. Patient questionnaires and behavioral histories were also surveyed during the 5 days ECG measurements, including the timing of bathing, presence of arrhythmic symptoms, and comfort of wearing the electrodes. Basic patient information is shown in Table 1.

**TABLE 1:** Patient background.

Age (years)	57 ± 17.4
Number of patients	294
Gender (Male/Female)	102/192
Indication	
Palpitations	210 (71.4%)
Lightheadedness	21 (7.14%)
Tachycardia	10 (3.40%)
Bradycardia	7 (2.38%)
Syncope	5 (1.70%)

*Note*: Age data are shown as means ± standard error of the mean (SEM). All percentages are presented as three significant digits.

### 2.2 eMEMO® recording and evaluation of noise contamination rate

**FIGURE 1:**
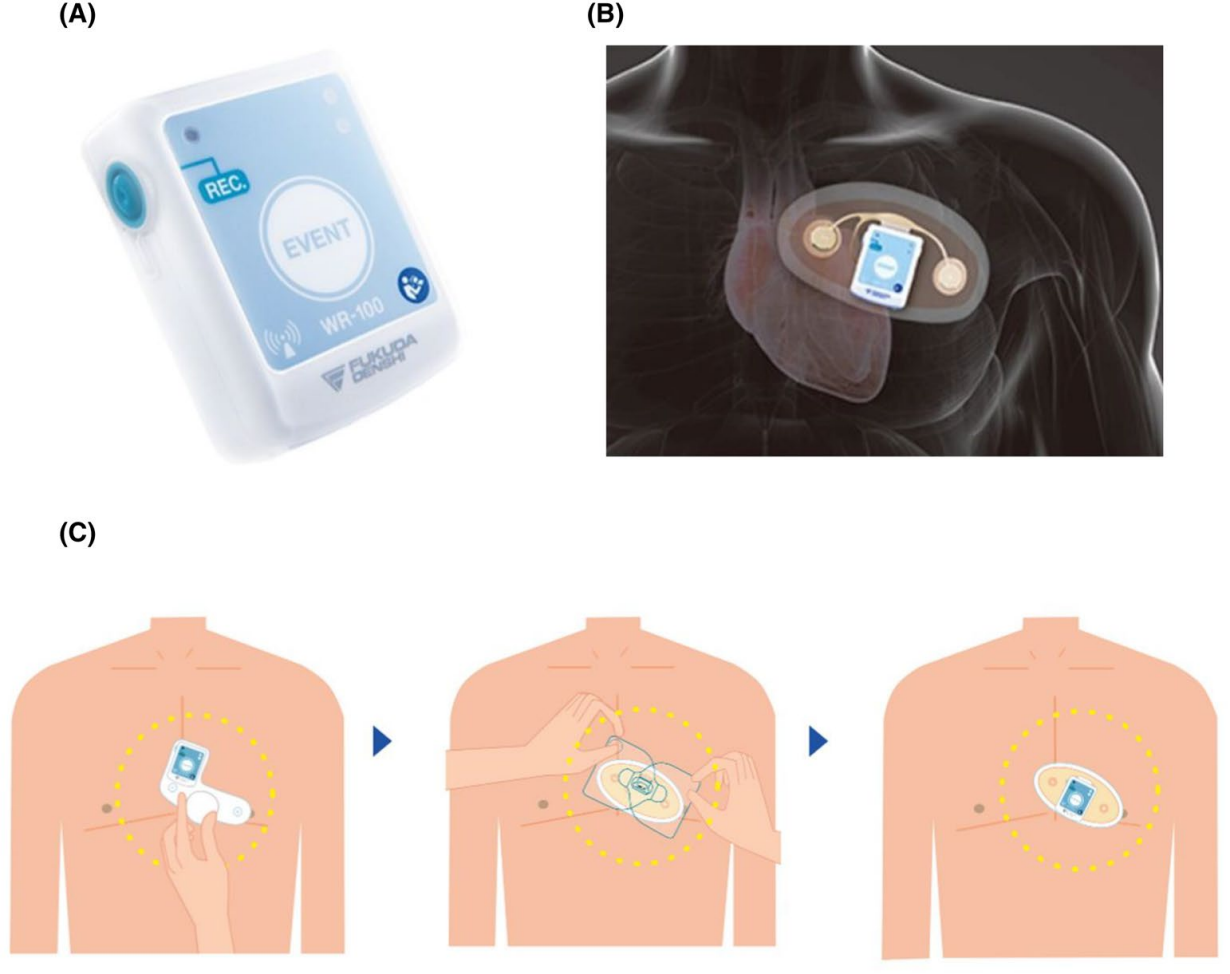
Appearance of eMEMO®. (A) Appearance of main unit (B) Appearance when attached to the body with a patch‐type electrode. (C) Optimal P wave detection before attaching the electrode. The electrical axis (electrode‐to‐electrode angle) where the largest and clearest P wave was recorded is checked using a dedicated device before the test begins, and a patch‐type electrode is attached at the optimal location. Source: Editing the product introduction from Fukuda Denshi Corp.; https://www.fukuda.co.jp/medical/icm/esus/longterm_electrocardiogram_examination_introduction.

**FIGURE 2:**
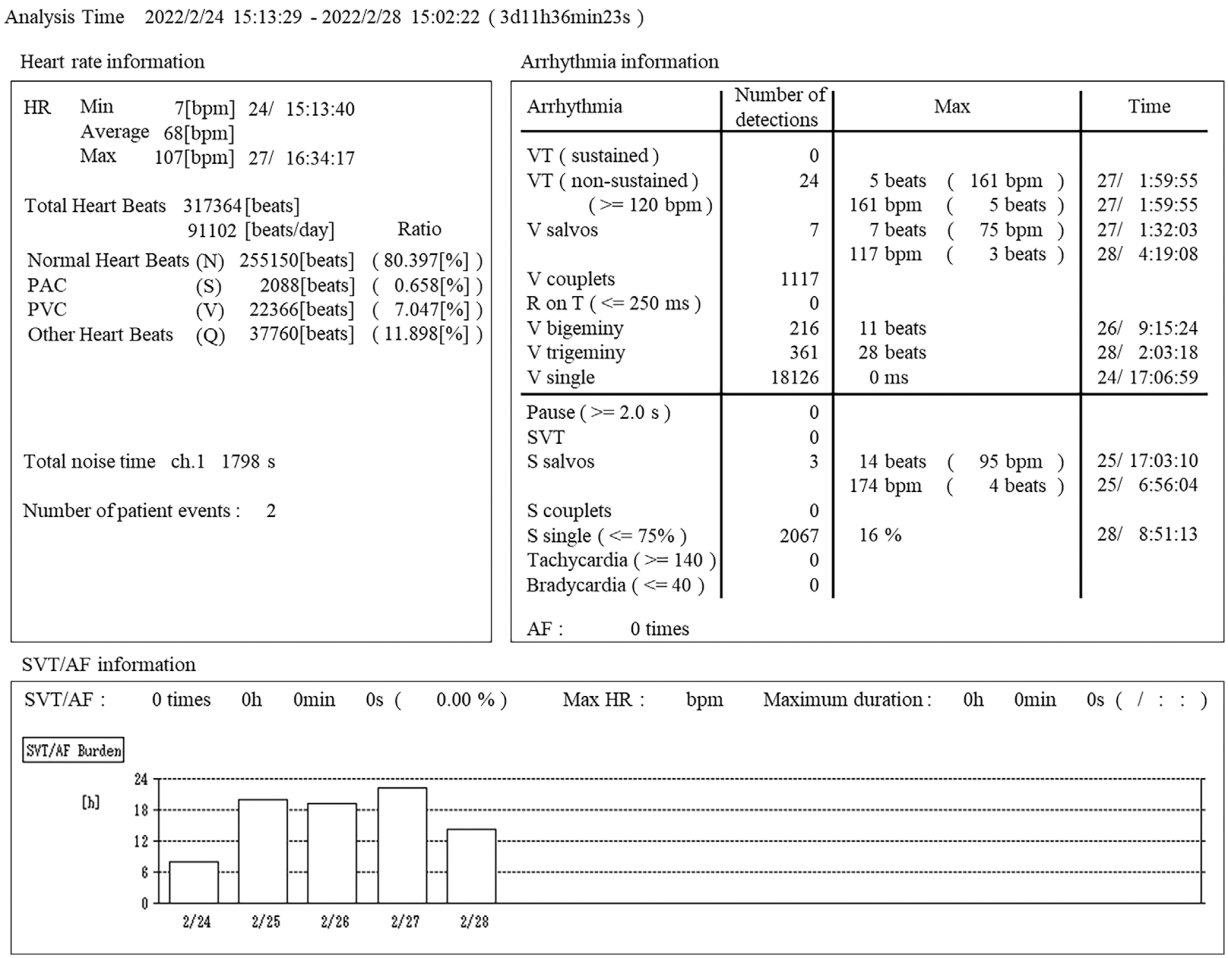
An example of actual eMEMO® analysis. Detailed data regarding heart rate, arrhythmia, and SVT/AF were automatically analyzed using dedicated software. AF, atrial fibrillation; PAC, premature atrial contraction; PVC, premature ventricular contraction; SVT, supraventricular tachycardia; VT, ventricular tachycardia.

**FIGURE 3:**
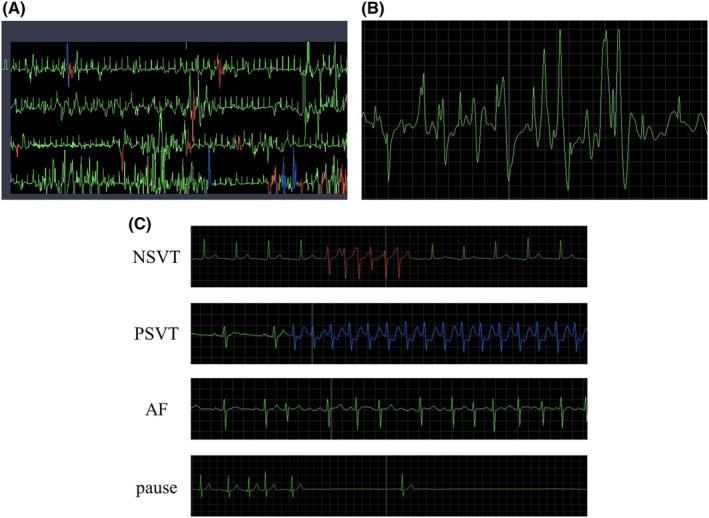
ECG waveform including obvious noise during measurement. (A) An example of ECG in a noisy area (B) Detailed ECG waveform in a noisy area. (C) The actual measured arrhythmia waveforms. NSVT, PSVT, AF, and pause were detected with good accuracy. AF, atrial fibrillation; NSVT, non‐sustained ventricular tachycardia; PSVT, paroxysmal supraventricular tachycardia.

eMEMO® is a single‐channel device (eMEMO® WR‐100, Fukuda Denshi, Tokyo, Japan) configured using a standardized protocol (Figure 1A,B). A distinct advantage of eMEMO® is that it can monitor and locate the electrode where p waves are most easily detected before attaching the electrode (Figure 1C). The eMEMO® electrode was attached by a trained clinical technologist at the location where the largest p wave can be measured. After 5 days of continuous recording, the ECG data were automatically sent to the analyzing PC via a USB connection. The collected ECG data were analyzed using analysis software (HPS‐100AF, Fukuda Denshi, Tokyo, Japan). Figure 2 shows an example of actual eMEMO® analysis. As shown in Figure 3A,B, the noise contamination rate was manually calculated in the total recording time. The noise contamination rate was calculated as follows: analysis exclusion time/total recording time × 100.

### 2.3 Automatic detection of arrhythmia

The automatic detection of arrhythmia was based on algorithms programmed by the manufacturer (Table 2). All waveforms were reviewed by skilled medical technologists at Taniguchi Heart Clinic and corrected for errors. A cardiologist then verified the results.

**TABLE 2:** Rhythm disturbances programmed for auto‐trigger detection of eMEMO®.

Rhythm disturbances	Programmed thresholds
V couplets	Two consecutive PVC
V salvos	Three or more consecutive PVC
VT	Three or more consecutive PVC and an average heart rate greater than the set value ※set range: 100–200 beats/minute
R on T	PVC occurs within a set time after the preceding normal R wave ※set time: 200–500 ms
V bigeminy	Normal heartbeat and PVC appear alternately
V trigeminy	Two consecutive normal heartbeats followed by the appearance of one PVC
Pause	R wave does not occur for more than a set time ※set time: 1.5–5.0 s
PAC	Early contraction occurs within a set value, with the mean value of the normal R‐R interval as 100% ※set value: 35%–90%
S couplets	Two consecutive PAC
S salvos	Three or more consecutive PAC
SVT	Three or more consecutive PAC and an average heart rate greater than the set value ※set range: 100–200 beats/minute
Tachycardia	Heart rate averages more than 3 consecutive beats above the set value ※set range: 100–200 beats/minute
Bradycardia	Heart rate averages more than 3 consecutive beats below the set value ※set range: 30–100 beats/minute

### 2.4 Detection of arrhythmic events during bathing

We instructed the participants, “You can bathe, but please use lukewarm water below 40°C for no more than 10 min and make sure that the body of the eMEMO® is not submerged in the water.” Based on the patients' behavioral history records, the presence or absence of arrhythmic events within 1 h from the start of bathing and their arrhythmia detection rates were investigated.

### 2.5 Comfort of wearing electrodes

During the long duration of eMEMO® measurement of up to 5 days, there were concerns that patients may feel some skin irritation, itching, and discomfort because of sweat during bathing or exercise. Therefore, 65 of the 294 patients who completed the questionnaire were surveyed and scored for^1^ how many days they felt itchy,^2^ itchiness,^3^ redness of the skin and rash, and^4^ comfort of the electrode. The evaluations of each item are presented in Table 3.

**TABLE 3:** The scoring foreach evaluation item.

Evaluation item	Detail	Score
How many days they felt itchy	No itching	0
	For 1 day	1
	For 2 days	2
	For 3 days	3
	For 4 days	4
	For 5 days	5
Itchiness	No itching	0
	Mild itching	1
	Moderate itching	2
	Severe itching	3
Redness of the skin and rash	No redness	0
	Mild erythema	1
	Erythema	2
	Erythema, Edema, Pimple	3
	Erythema, Edema, Pimple, Blister	4
Comfort of the electrode	Comfortable	0
	Mostly comfortable	1
	A little uncomfortable	2
	Uncomfortable	3

## 3 RESULTS

### 3.1 Arrhythmia detection

Figure 3C shows the actual arrhythmia waveforms. Arrhythmogenic ECG waveforms were clearly acquired without being affected by the noise caused by daily activities, exercise, and bathing. The arrhythmia detection results for all 294 patients are presented in Table 4. Premature ventricular contraction (PVC) was detected in 260 patients and premature atrial contraction (PAC) in 285 patients during the 5 days, while other arrhythmias were detected in 31 patients with non‐sustained ventricular tachycardia/sustained ventricular tachycardia (NSVT/SVT), 56 patients with paroxysmal supraventricular tachycardia/paroxysmal atrial tachycardia (PSVT/PAT), 23 patients with AF, 41 patients with a pause longer than 2 s, and 6 patients with atrioventricular block (AVB) of degree II or higher. In addition, among these arrhythmias, NSVT/SVT (58.1%), PSVT/PAT (57.1%), AF (34.8%), a pause longer than 2 s (51.2%), and AVB (50.0%) were the first detected after 24 h of recording.

**TABLE 4:** Detection of arrhythmia within and over 24 h using eMEMO® devise.

	Total	Detect within 24 h	Detect over 24 h
Number of patients	294	165	127 (43.2%)
Number of total Arrhythmias	799	629	170 (21.3%)
PVC	260	210	50 (19.2%)
Ventricular bigeminy	44	29	15 (34.1%)
Ventricular trigeminy	53	41	12 (22.6%)
NSVT/SVT	31	13	18 (58.1%)
PAC	285	274	11 (3.86%)
PSVT/PAT	56	24	32 (57.1%)
AF	23	15	8 (34.8%)
Pause >2 s	41	20	21 (51.2%)
AVB (>II degree)	6	3	3 (50.0%)

*Note*: All percentages are shown as 3 significant digits.

Abbreviations: AF, atrial fibrillation; AVB, atrioventricular block; NSVT, non‐sustained ventricular tachycardia; PAC, premature atrial contraction; PAT, paroxysmal atrial tachycardia; PSVT, paroxysmal supraventricular tachycardia; PVC, premature ventricular contraction; SVT, sustained ventricular tachycardia.

### 3.2 Evaluation of noise contamination rate

**FIGURE 4:**
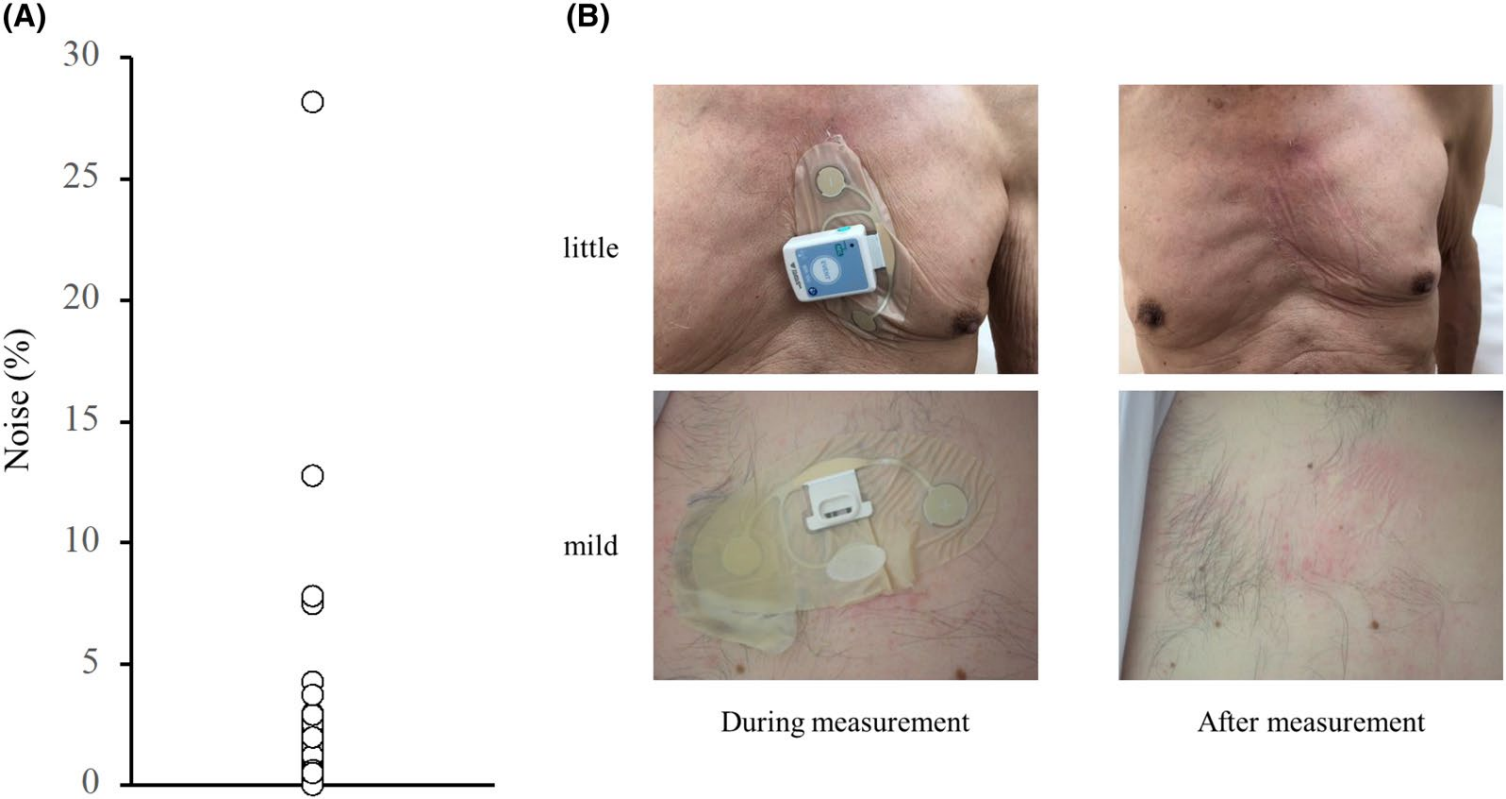
The skin condition varied from patient to patient, with many patients having good skin conditions with little or no redness; however, some patients had mild skin conditions such as redness, itching, and eczema. (A) The noise contamination rate for all 294 patients over the 5 days. Of the 294 patients, 290 (98.6%) had a noise contamination rate of only 5% or less. (B) Skin condition during and after measurement.

Although there were concerns regarding noise increase because of degradation of the patch electrode during long‐term measurement over a period of up to 5 days, the noise contamination rate calculated from total noise, which was excluded from the analysis in the total measurement time, was very low at 0.50 ± 2.0%, demonstrating that stable recording was possible. Figure 4(A) shows the variation in the noise contamination rate for all 294 patients over the 5 days. Of the 294 patients, 290 (98.6%) had a noise contamination rate of only 5% or less, indicating that extremely stable ECG recording was possible. On the contrary, four patients (1.4%) had a high noise contamination rate of 7%–28%, making analysis difficult. In fact, when we checked the ECG waveforms of those with a high noise contamination rate, the P and QRS waves were difficult to read, and automatic detection by the analysis software was impossible for several hours (Figure 3A,B).

### 3.3 Detection of arrhythmic events during bathing

Arrhythmia detection within 1 h of the start of bathing was examined for all 69 patients who completed the questionnaire regarding bathing time, with 52 patients experiencing arrhythmic events during bathing. Of the 52 patients, 17 had subjective symptoms, and 35 had no symptoms. The subjective symptoms and types of arrhythmias in all 17 patients are shown in Table 5. The most common subjective symptoms were palpitations, difficulty breathing, and chest pain (76.5%), with a few patients complaining of dizziness, skin tightness, or others. The types of arrhythmias were AF in 2 patients (11.8%), PAC in 8 patients (47.1%), and PVC in 7 patients (41.2%).

**TABLE 5:** The subjective symptoms and type of arrhythmia during 1 h from the start of bathing.

Indication	Type of arrhythmia
Palpitation	AF, AFL
Palpitation	Tachycardia
Palpitation	PAC
Palpitation	PAC
Palpitation	PVC
Palpitation	PVC
Palpitation	PVC
Difficulty breathing	PAC, PVC, Tachycardia
Difficulty breathing	PAC
Difficulty breathing	PAC
Difficulty breathing	PVC
Difficulty breathing	PVC
Chest pain	PVC
Dizziness	PAC
Skin tightness	PAC
Disturbed pulse	AF
Tachycardia	PAC

Abbreviations: AF, atrial fibrillation; AFL, atrial flutter; PAC, premature atrial contraction; PVC, premature ventricular contraction.

### 3.4 Skin condition when removing the patch‐type electrode after 5 days of monitoring

**FIGURE 5:**
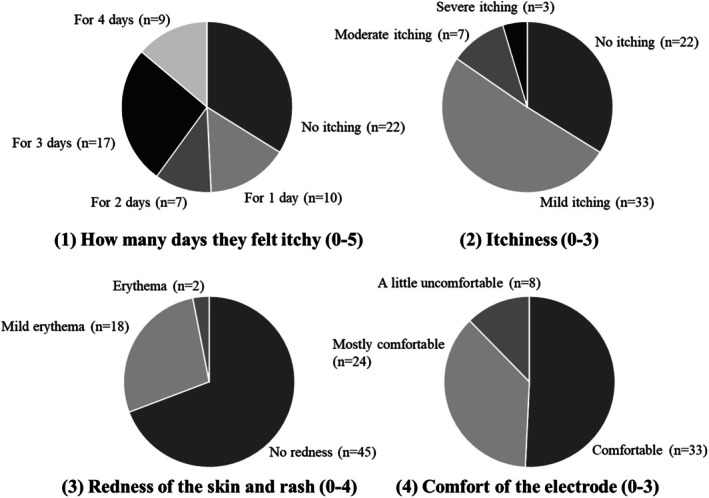
Results of a survey on four skin condition assessments (*n* = 65). Mild erythema occurred in approximately 30.8% of patients, but most of them (87.7%) reported that the patch electrode was comfortable to wear for a long period of time.

The skin condition at the time of patch electrode removal after 5 days of measurement is shown in Figure 4B. The skin condition varied from patient to patient, with many patients having good skin conditions with little or no redness; however, some patients had mild skin conditions such as redness, itching, and eczema. In fact, the patient questionnaire showed that the largest number of patients (*n* = 22) did not feel itching throughout the 5 days, and most patients evaluated the skin itching level as none to mild (Figure 5). Mild erythema occurred in approximately 30.8% of patients, but most of them (87.7%) reported that the patch electrode was comfortable to wear for a long period of time. Many of the free‐writing comments were positive, such as comfort with less itching and rash, no complications in putting on and taking off, and electrode cords not obtrusive. However, there were also some negative comments, such as the difficulty of pressing the event button when wearing clothes, and the patch tape easily peeled off when taking a bath.

## 4 DISCUSSION

In this study, we took advantage of the characteristics of a novel compact external loop recorder (eMEMO®) with waterproofing, long‐term monitoring, and automatic arrhythmia detection to investigate its usefulness for long‐term recording over a period of up to 5 days and for ELR measurement during bathing. The advantages of eMEMO® are discussed in five sections.

### 4.1 Automatic recording and long‐term measurement

It is generally known that the detection of various types of arrhythmias, including atrial fibrillation, increases with recording time. Locati et al. performed long‐term longitudinal event‐recorder ECG monitoring in patients with persistent palpitations of unknown cause and found that the detection rates of the arrhythmias were 42.4% at 1 week, 57.2% at 2 weeks, and 71.6% at 4 weeks. The detection rate was 50% at 1 week and 70% at 2 weeks in patients who had previously experienced frequent palpitations or were investigated quickly after subjective symptoms.^12^ In our study where ECG was recorded for up to 5 days, approximately 71% of the patients had palpitations as the main symptom. Recordings for longer than 24 h led to the first arrhythmia detection in 170 cases (21.3%) out of the 799 cases of all detected arrhythmias. In particular, NSVT/SVT, PSVT/PAT, and pause were detected for the first time after 24 h in more than half of the patients (Table 4), giving an arrhythmia detection rate comparable to that of previous studies. This is likely because the eMEMO® has a highly accurate automatic detection of arrhythmias. Among the 16 patients who underwent long‐term ELR testing with eMEMO® for longer than 10 days during the same study period, no new arrhythmias were detected after day 6 of testing (the arrhythmia detection rate plateaued after the initial 5 days of testing). Comparing two ELRs (SpiderFlash®) with and without automatic detection in patients with palpitations, the former detected more asymptomatic arrhythmias, mainly pauses, bradycardia, AVB, and sustained paroxysmal atrial fibrillation (PAF)/PSVT/NSVT, and was suggested to have a superior ability to capture temporary rhythm disturbances.^13^ The automatic detection function of eMEMO® is similar to that of the SpiderFlash® algorithm (Table 2), which could have recorded transient arrhythmias with high accuracy. However, the disadvantage of conventional ELRs is that they cannot record continuously and require the pressing of an event button when symptoms occur. Sivakumaran et al. reported that one‐quarter of patients who used the ELR did not press the event button despite having been given prior instructions.^14^ Automatic detection of arrhythmia has been reported to be very effective, and there are many cases in which asymptomatic arrhythmias were efficiently detected.^13^, ^15^ In this study, we identified several cases of asymptomatic arrhythmias that were not recorded in the event button or written in the action record. These suggest the usefulness of the long‐term recording and automatic detection in eMEMO®.

### 4.2 Determine the electrode position where the p wave is maximum before monitoring

The accuracy of automated analysis of ECG data is extremely important in the diagnosis of arrhythmias over long periods of time, because the visual confirmation that has traditionally been performed is time‐consuming and may miss arrhythmias. The setting conditions of automatic recording significantly affect the diagnosis or analysis, and its accuracy is significantly influenced by the noise in the ECG waveform. In fact, a survey conducted at Toho University Medical Center in 2022 reported that although automatic recording has a high arrhythmia detection capability, it also has a high rate of false determinations owing to noise and other factors. Even though an implantable loop recorder (ILR) which is considered less affected by noise and more sensitive than an ELR, when 85 patients were labeled with AF by automatic detection, 43 of them actually had AF and 42 of them (48.3%) were mislabeled.^16^ Since the automatic ECG diagnostic system using computers was first introduced, recognition of the P wave has been considered an important factor that greatly influences diagnosis. The P wave is difficult to recognize because of its small amplitude, slow waveform, and large fluctuations. However, it is especially important to obtain a clear P wave recording because tachyarrhythmias are classified according to the P wave. In eMEMO®, the electrical axis (electrode‐to‐electrode angle) where the P wave is clearly recorded and has the largest amplitude can be confirmed before starting the clinical examination using a dedicated device (Figure 1C). Various types of ELR, including SpiderFlash®, a type of ELR conventionally used around the world, are unable to detect such electrical axis, making automatic analysis impossible and leading to incorrect diagnosis in many cases. In this study, AF was detected in 23 patients. Although P waves were clearly visible, a few false‐positive findings were registered as AF; however, no false‐negative findings occurred. While no previous reports have evaluated the automatic diagnosis of eMEMO®, a study examining the accuracy of AF detection using another ELR (R. Test Evolution 4) reported a sensitivity of 84% and a specificity of 98%.^17^ Similarly, a study using the ELR Vitaphone 3100 BT found a sensitivity of 93% and a specificity of 51%.^18^ The ability to confirm the P waveform before starting the clinical examination can be a major advantage that is unique to eMEMO®.

### 4.3 Low noise contamination rate

Artifacts, such as noise, greatly affect the quality of ECG during long‐term monitoring. Although good‐quality ECG recording without noise is required for proper arrhythmia diagnosis, in conventional ELR and 24‐h Holter ECG, artifacts because of bad connection of the electrode leads of the recorder itself are contaminated, in addition to electromyography or respiratory baseline perturbations. Hotta et al. reported that the most common cause of artifact contamination of ECG monitors in hospitalized patients is electrode dropout or poor contact, and that electrode cord snagging or pulling is also likely to result in poor contact. The eMEMO® uses a patch‐type electrode that can be taped together with the cord and the ECG main unit in one piece, making it less likely to detach from the electrode than conventional devices and more resistant to noise from daily activities (Figure 4(A)). The noise contamination rate (analysis exclusion time/total recording time × 100) was calculated for all patients. Out of the total 294 patients, 290 had a noise contamination rate of 5% or less, indicating stable recording. In the four patients with noise contamination rates exceeding 5%, the cause was obvious as the patient's activity record chart showed that the electrode seal had been largely detached during the recording. Furthermore, all 16 patients who underwent testing with eMEMO® for longer than 10 days had a noise contamination rate of less than 5%. A study using other patch‐type electrodes (AT‐patch) reported that the average noise time was 15.8% of the total ECG recording time.^19^ This indicates that the eMEMO® provides more stable ECG recording. After recording the ECG continuously for up to 5 days, the patch‐type electrode was found to be detached in 15 of the 294 patients, and in only the above four patients was it so severe that noise was contained in the ECG waveform. A patch‐type electrode fixed together with the cord and ECG main unit can improve the diagnostic accuracy of the ECG by reducing the noise contamination rate.

### 4.4 Waterproof function and record during bathing

It has long been well known that sympathetic activation is likely to cause malignant arrhythmias.^20^ It is generally believed that during bathing, the parasympathetic nervous system becomes dominant, leading to a decrease in blood pressure and cardiac workload. However, high‐temperature bathing of 42°C or higher stimulates the sympathetic nervous system, causing blood pressure and heart rate to rise quickly and place a heavy burden on the circulatory system. This disturbance of the autonomic nervous system and fluctuations in blood pressure can easily induce dangerous arrhythmias during bathing, especially during high‐temperature bathing, which is a unique Japanese habit. In fact, there has been a rapid increase in cardiovascular events during bathing, especially in the elderly, who are strongly suspected to have ischemic heart disease,^10^ and has been called “Death‐by‐bathing syndrome for the elderly” etc. Therefore, it is useful to examine ECG changes during bathing, but most 24‐h Holter ECG and SpiderFlash® cannot record during bathing. In contrast, eMEMO® has a waterproof and cordless design with patch‐type electrode, allowing continuous recording during bathing with minimal discomfort. In the present study, arrhythmias were detected in 17 patients with subjective symptoms during bathing and 35 patients without symptoms. AF or atrial flutter (AFL) were found in addition to the commonly detected PAC or PVC (Table 5). Therefore, the ability to detect arrhythmias continuously while bathing with minimal discomfort is of great clinical importance and is a major advantage of the eMEMO®.

### 4.5 Less itchy skin even after prolonged wearing

Although long‐term ECG recording is beneficial for detecting arrhythmias, especially for ELR devices with chest electrodes, comfort during electrode attachment is crucial. The prolonged wear of heavy devices with electrodes and skin reactivity to the electrodes may affect patient comfort. In a study using SpiderFlash®, which is a commonly used ELR worldwide, 3% of patients had some skin discomfort from the electrodes even in recording periods of less than 2 days in some cases.^12^ Of the 395 study subjects, 4 had their ECG recordings interrupted because of discomfort with the electrodes, and two dropped out. In a 7‐day testing using another ELR device (R.Test Evolution 4®), 17 of the 217 patients (7.8%) removed the device within 2 days because of electrode‐induced skin irritation.^21^ In our recommended ELR electrocardiograph eMEMO®, none had skin problems that prevented continuous recording during the 5 days. Although 27 of the 481 patients (5.6%) developed a rash during the 7‐day testing with the R‐Test ELR device,^22^ only skin erythema was observed in our study, with no patients developing a rash. Mild erythema occurred in 6 of the 16 patients (37.5%) who underwent testing with eMEMO® for longer than 10 days, which was not significantly different from the rate observed during the 5 days testing (30.8%). Regarding the skin itching level, 13 of the 16 patients (81.3%) evaluated it as none to mild, with no change after the 5 days testing (84.6%). Furthermore, in a report on 7‐day testing using electrodes designed to minimize rash in SpiderFlash®, 29% of the participants experienced severe itching (compared to 4.6% with eMEMO®), 69% developed skin erythema (30.8% with eMEMO®), and 18.6% developed blisters (0% with eMEMO®). This patch‐type electrode has a smaller contact area with the skin and is made of a skin‐friendly material that is thinner and more flexible than conventional electrode tape; therefore, most patients experience little redness and only mild itching when the electrode is removed (Figures 4B and 5).

## 5 LIMITATIONS

In the present study, the maximum duration of recording was 5 consecutive days, but it is not clear whether this was the optimal period for detecting arrhythmias. Locati et al. reported that in patients with palpitations of unknown cause, ECG monitoring for up to 4 weeks is considered the best diagnostic tool and provides a definitive diagnosis in most patients.^12^ Akca et al. concluded that the optimal recording time for arrhythmia detection in children is 2 weeks, considering diagnostic efficiency and cost‐effectiveness.^23^ In this experiment, however, measurements were performed for a maximum of 5 days, taking into consideration the overall burden of prolonged electrode wear on patients, detection efficiency, cost‐effectiveness, and other factors. In fact, supporting evidence is available in the literature. For example, in a study using a different patch‐type electrode in patients experiencing symptoms suggestive of arrhythmia, the median time to first detection of significant arrhythmias was 3.1 days for VT, 4.2 days for pause, and 5.8 days for AVB, suggesting that approximately 5 days of ECG monitoring may be effective.^24^ Similarly, a study using a continuous loop recorder to examine arrhythmias in outpatients with symptoms such as palpitations, dizziness, or syncope reported that 75% of patients received a diagnosis after a mean ECG monitoring duration of 5.2 ± 2.3 days.^25^ While long‐term ECG monitoring, such as over 2 weeks, is necessary to detect PAF, clinical practice often emphasizes the efficient detection of general arrhythmias, not just PAF. Therefore, a 5‐day monitoring period was adopted based on this rationale.

## 6 CONCLUSION

The patch‐type ELR (eMEMO®), which is waterproof and can continuously record regardless of restrictions such as bathing or exercise, is useful for detecting stable arrhythmias in patients.

## FUNDING INFORMATION

There is no funding source.

## CONFLICT OF INTEREST STATEMENT

The authors have no conflict of interest to declare.

## APPROVAL OF THE RESEARCH PROTOCOL

Human participants were involved in this study.

## REGISTRY AND THE REGISTRATION NO. OF THE STUDY/TRIAL

2211‐030.

## References

[1] Choi SE , Sagris D , Hill A , Lip GYH , Abdul‐Rahim AH . Atrial fibrillation and stroke. Expert Rev Cardiovasc Thre. 2023;21(1):35–56. 10.1080/14779072.2023.2160319 36537565

[2] Camm AJ , Lip GY , De Caterina R , Savelieva I , Atar D , Hohnloser SH , et al. 2012 focused update of the ESC guidelines for the management of atrial fibrillation: an update of the 2010 ESC guidelines for the management of atrial fibrillation. Developed with the special contribution of the European heart rhythm association. Eur Heart J. 2012;33(21):2719–2747. 22922413 10.1093/eurheartj/ehs253

[3] Zoni‐Berisso M , Lercari F , Carazza T , Domenicucci S . Epidemiology of atrial fibrillation: European perspective. Clin Epidemiol. 2014;6:213–220. 24966695 10.2147/CLEP.S47385 PMC4064952

[4] Kennedy HL . The evolution of ambulatory ECG monitoring. Prog Cardiovasc Dis. 2013;56(2):127–132. 24215744 10.1016/j.pcad.2013.08.005

[5] Kwon S , Lee SR , Choi EK , Ahn HJ , Song HS , Lee YS , et al. Comparison between the 24‐hour Holter test and 72‐hour single‐Lead electrocardiogram monitoring with an adhesive patch‐type device for atrial fibrillation detection: prospective cohort study. J Med Internet Res. 2022;24(5):e37970. 35532989 10.2196/37970 PMC9127648

[6] Chua SK , Chen LC , Lien LM , Lo HM , Liao ZY , Chao SP , et al. Comparison of arrhythmia detection by 24‐hour Holter and 14‐day continuous electrocardiography patch monitoring. Acta Cardiol Sin. 2020;36(3):251–259. 32425440 10.6515/ACS.202005_36(3).20190903A PMC7220965

[7] Mittal S , Movsowitz C , Steinberg JS . Ambulatory external electrocardiographic monitoring: focus on atrial fibrillation. J Am Coll Cardiol. 2011;58(17):1741–1749. 21996384 10.1016/j.jacc.2011.07.026

[8] Gambino A , Ravetti E , Naldi A , Russo R , Molinaro S , Mistretta F , et al. Embolic stroke of undetermined source: role of implantable loop recorder in secondary prevention. Can J Neurol Sci. 2023;50(4):529–534. 35656581 10.1017/cjn.2022.66

[9] Sharma AN , McIntyre WF , Nguyen ST , Baranchuk A . Implantable loop recorders in patients with atrial fibrillation. Expert Rev Cardiovasc Ther. 2022;20(12):919–928. 36444859 10.1080/14779072.2022.2153673

[10] Abe H , Kohno R , Oginosawa Y . Characteristics of syncope in Japan and the Pacific rim. Prog Cardiovasc Dis. 2013;55(4):364–369. 23472772 10.1016/j.pcad.2012.11.008

[11] Akbarzadeh A , Akbarzadeh F , Kazemi B . Simultaneous beat‐to‐beat heart rate and systolic blood pressure variability in patients with and without neurally mediated syncope. J Cardiovasc Thorac Res. 2022;14(2):108–115. 35935387 10.34172/jcvtr.2022.18 PMC9339730

[12] Locati ET , Moya A , Oliveira M , Tanner H , Willems R , Lunati M , et al. External prolonged electrocardiogram monitoring in unexplained syncope and palpitations: results of the SYNARR‐flash study. Europace. 2016;18(8):1265–1272. 26519025 10.1093/europace/euv311 PMC4974630

[13] Locati ET , Vecchi AM , Vargiu S , Cattafi G , Lunati M . Role of extended external loop recorders for the diagnosis of unexplained syncope, pre‐syncope, and sustained palpitations. Europace. 2014;16(6):914–922. 24158255 10.1093/europace/eut337

[14] Sivakumaran S , Krahn AD , Klein GJ , Finan J , Yee R , Renner S , et al. A prospective randomized comparison of loop recorders versus Holter monitors in patients with syncope or presyncope. Am J Med. 2003;115(1):1–5. 12867227 10.1016/s0002-9343(03)00233-x

[15] Müller A , Scharner W , Borchardt T , Och W , Korb H . Reliability of an external loop recorder for automatic recognition and transtelephonic ECG transmission of atrial fibrillation. J Telemed Telecare. 2009;15(8):391–396. 19948705 10.1258/jtt.2009.090402

[16] Kim SE , Khawaja M , Kim JA , Safavi‐Naeini P , Pickett J , Molina‐Razavi J , et al. Detection of atrial fibrillation in real world setting in patients with cryptogenic stroke and an implantable loop recorder. Pacing Clin Electrophysiol. 2023;46(7):788–795. 37323035 10.1111/pace.14757

[17] Sejr MH , May O , Damgaard D , Sandal BF , Nielsen JC . External continuous ECG versus loop recording for atrial fibrillation detection in patients who had a stroke. Heart. 2019;105(11):848–854. 30898849 10.1136/heartjnl-2018-314186

[18] Velthuis BO , Bos J , Kraaier K , Stevenhagen J , van Opstal JM , van der Palen J , et al. Performance of an external transtelephonic loop recorder for automated detection of paroxysmal atrial fibrillation. Ann Noninvasive Electrocardiol. 2013;18(6):564–570. 24303971 10.1111/anec.12075 PMC6932654

[19] Kwun JS , Lee JH , Park BE , Park JS , Kim HJ , Kim SH , et al. Diagnostic value of a wearable continuous electrocardiogram monitoring device (AT‐patch) for new‐onset atrial fibrillation in high‐risk patients: prospective cohort study. J Med Internet Res. 2023;25:e45760. 37721791 10.2196/45760 PMC10546264

[20] Vaseghi M , Shivkumar K . The role of the autonomic nervous system in sudden cardiac death. Prog Cardiovasc Dis. 2008;50(6):404–419. 18474284 10.1016/j.pcad.2008.01.003 PMC2752648

[21] Højager A , Tingsgaard JK , Andersen D , Søholm H , Taskiran M , Bock TG , et al. Silent atrial fibrillation detected by home‐monitoring: cardiovascular disease and stroke prevention in patients with diabetes. J Diabetes Complications. 2020;34(12):107711. 32900590 10.1016/j.jdiacomp.2020.107711

[22] Murphy R , Waters R , Murphy A , McDermott S , Reddin C , Hernon O , et al. Risk‐based screening for the evaluation of atrial fibrillation in general practice (R‐BEAT): a randomized cross‐over trial. QJM. 2025;118(3):166–173. 39786890 10.1093/qjmed/hcaf001 PMC12051387

[23] Akca T , Uysal F , Bostan OM , Genc A , Turkmen H . The role of external loop recorders in arrhythmia‐related symptoms in children: a single center experience. Pediatr Cardiol. 2022;43(1):147–154. 34389905 10.1007/s00246-021-02705-y

[24] Schreiber D , Sattar A , Drigalla D , Higgins S . Ambulatory cardiac monitoring for discharged emergency department patients with possible cardiac arrhythmias. West J Emerg Med. 2014;15(2):194–198. 24672611 10.5811/westjem.2013.11.18973 PMC3966438

[25] Martinez T , Sztajzel J . Utility of event loop recorders for the management of arrhythmias in young ambulatory patients. Int J Cardiol. 2004;97(3):495–498. 15561338 10.1016/j.ijcard.2003.11.004

